# Current concepts on *Pseudomonas aeruginosa* interaction with human airway epithelium

**DOI:** 10.1371/journal.ppat.1011221

**Published:** 2023-03-30

**Authors:** Anaëlle Muggeo, Christelle Coraux, Thomas Guillard

**Affiliations:** 1 Université de Reims Champagne-Ardenne, INSERM, CHU de Reims, Laboratoire de bactériologie-Virologie-Hygiène hospitalière-Parasitologie-Mycologie, P3Cell, U 1250, Reims, France; 2 Université de Reims Champagne-Ardenne, INSERM, P3Cell, U 1250, Reims, France; University of Basel, SWITZERLAND

## Abstract

*Pseudomonas aeruginosa* is a major, but opportunistic, respiratory pathogen, which rarely infects healthy individuals, mainly due to the barrier effect of the human airway epithelium (HAE). This review explores the interaction of *P*. *aeruginosa* with HAE and the progression of the infection. The basolateral part of the epithelium, which includes the basolateral membrane of the epithelial cells and the basement membrane, is inaccessible in normal tight epithelia with intact junctions. We highlight how *P*. *aeruginosa* exploits weaknesses in the HAE barrier to gain access to the basolateral part of the epithelium. This access is crucial to initiate respiratory infection and is mainly observed in the injured epithelium, in repairing or chronically remodeled epithelium, and during extrusion of senescent cells or cell multiplication during normal epithelium renewal. The subsequent adhesion of the bacteria and cytotoxic action of virulence factors, including the toxins delivered by the type 3 secretion system (T3SS), lead to retractions and cell death. Eventually, *P*. *aeruginosa* progressively reaches the basement membrane and propagates radially through the basal part of the epithelium to disseminate using twitching and flagellar motility.

## Introduction

*Pseudomonas aeruginosa* is a ubiquitous gram-negative bacterium mainly found in aqueous environments and surfaces [1–4]. This opportunistic pathogen is responsible for various infections, particularly those involving the respiratory tract [5–7]. Owing to intact airway epithelial barrier [8–11] and optimal lung defenses, *P*. *aeruginosa* is rarely pathogenic in healthy individuals [12], and the development of infections depends on epithelial injuries [13–16], mucociliary clearance dysfunctions [17], or immune system impairment [18–21]. Acute or chronic pneumonia is limited to immunocompromised patients or those with defective pulmonary function, such as mechanically ventilated patients in intensive care unit [5,22,23], and those with cystic fibrosis (CF) [24,25] or chronic obstructive pulmonary disease (COPD) [7,26–28].

**Fig 1:**
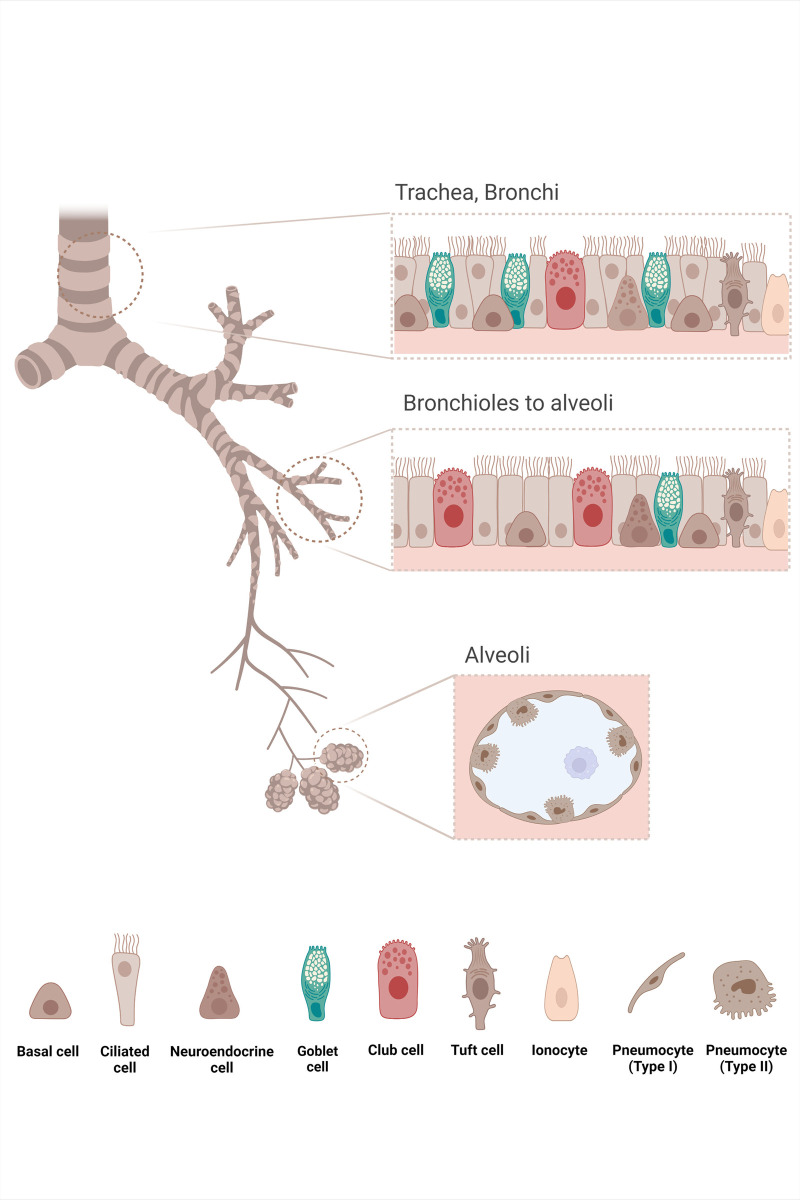
Lung epithelia structure. The lung is a complex organ composed of conducting airways and gas exchange zones. The conducting airways branch from the trachea to terminal bronchioles that end up in the alveoli. In airways, the lining epithelium provides a physical barrier between the external environment and the underlying parenchyma, whose integrity is maintained by intercellular junctions. The airway epithelium ensures the protection of the lung against inhaled particles, toxins, and pathogens through the mucociliary clearance and secretion of molecules with antibacterial, antioxidant, and antiprotease activity that act in an orchestrated way to protect the epithelium from lesion factors. The diversity of cells constituting the epithelial barrier is adapted to the epithelial functions. In the pseudostratified epithelium lining the trachea and bronchi, different specific cell types such as basal, goblet, and ciliated cells are found. Basal cells are progenitor cells involved in the epithelial renewal and the anchor of the epithelium to the basement membrane through hemidesmosomes. Goblet cells, within subepithelial glands, produce the respiratory mucus that entraps noxious particles and is moved towards the oropharyngeal junction by the coordinated beating of ciliated cells. Some rare secretory Club cells are also described. In the distal bronchioles, the number of basal cells decreases, and the goblet cells are progressively replaced by Club cells, mainly involved in the production of anti-inflammatory factors and surfactant proteins. In addition, other cell types were found more rarely: neuroendocrine cells, which serve as communicators between the immune and nervous system by secreting neuropeptides; Tuft cells, which have chemosensory, neuronal, and immunological functions; and pulmonary ionocytes. The alveoli are lined by type I and type II pneumocytes whose functions are completely different: Type I pneumocytes are involved in the O_2_/CO_2_ exchange through their thin cytoplasm, whereas type II pneumocytes secrete surfactant proteins and act as progenitor cells of the alveolar epithelium. Created with BioRender.com.

The intact human airway epithelium (HAE) is a physical and functional barrier against pathogens [29,30]. The pseudostratified and differentiated mucociliary bronchial epithelium is usually composed of several different types of cells, supported by the underlying basement membrane [31] (Fig 1). The ciliated and secretory cells, which are tall, functionally differentiated, and highly polarized, reach the apical surface. The basal cells, which are located at the basal pole under the columnar cells, are multipotent progenitors, which generate the other cells during the differentiation process [31–33]. In intact healthy HAE, only apical membranes are exposed to the environment [20,34]. Injuries, varying from junction disruptions to partial shedding of the epithelium, or even to complete denudation of the basement membrane, induce the HAE to initiate a repair process to regenerate and restore its function [35–37]. In chronic inflammatory respiratory diseases such as CF or COPD, the dysregulated inflammation can alter the respiratory epithelial repairing process [14,15,35,38–42].

The airway epithelium protects the lungs from various external assaults (pollutants, pathogens, allergens, etc.) in three ways. First, the airway epithelium is a physical barrier. Its impermeability is ensured by strongly connected cells by tight junctions, adherens junctions, and desmosomes [43,44]. The tight junctions, located near the apical border, delimit the apical plasma membrane from the basolateral plasma membrane of the differentiated cells [45–47]. In healthy epithelium, the basolateral part, which is composed of the basolateral cell membrane and the underlying basement membrane, is not exposed to external agents [29,43]. Second, the airway epithelium can expel inhaled particles from the airways using the mucociliary clearance mechanism. The mucociliary clearance is carried out by the ciliated and secretory cells. It is the main process for removing inhaled foreign particles from the airways [44]. The airway surfaces are lined by epithelial cells and covered with an airway surface layer (ASL) composed of two parts: a mucus layer and a low-viscosity periciliary layer (PCL). Thus, the ASL lubricates airway surfaces and facilitates ciliary beating for efficient mucus clearance [17]. The mucus produced by secretory cells forms a continuous layer on the epithelial surface, and this three-dimensional matrix acts as a physical barrier protecting the underlying epithelia and as a trap for inhaled particles and exogenous microorganisms [48–50]. It is composed of mucins (MUC5ac and MUC5b), which are heavily glycosylated proteins within the mucus to which the pathogens attach [51–53]. Thereby, the coordinated beating of the ciliated cells’ cilia sweeps the trapped bacteria out of the lungs toward the oropharynx where they are swallowed. Third, the airway epithelium is capable of defending itself against infectious agents. The airway epithelium can produce antimicrobial peptides and inflammatory cytokines, which participate in the innate immune response [20,29].

This review discusses the interaction of *P*. *aeruginosa* with HAE as it is an integral part of its virulence in the respiratory tract. We will first highlight that the essential step to initiate airway infection is access to the basolateral part of the HAE by *P*. *aeruginosa*. Then, we will identify the different steps in the HAE infection by *P*. *aeruginosa* and discuss some specific issues, such as how the bacteria adhere to the HAE cells, what is the role of pathogen internalization by HAE cells in infection, and how *P*. *aeruginosa* crosses and invades the epithelium.

### Access to the basolateral part of HAE is crucial for *P*. *aeruginosa* to initiate respiratory infections

#### 1) Breach of the epithelium is a hallmark of *P*. *aeruginosa* infections

The HAE plays a key role in defense against *P*. *aeruginosa* by being a functional physical barrier thanks to intercellular junctions, mainly tight junctions, leading to epithelial polarity and impermeability. The ability to exploit epithelial breaches to reach the binding receptors in deeper tissues is a hallmark of *P*. *aeruginosa* infections. Most, if not all, pathologies caused by this bacterium illustrate this point. Cutaneous infections mostly occur after skin injuries such as extended burns or chronic cutaneous wounds, the latter being quite common among diabetic patients [54,55]. Keratitis or corneal infections usually follow disruption of the corneal epithelium occurring after ocular trauma, surgery, or lesions caused by contact lenses [56–59]. The risk factors of acute otitis externa, which is mainly observed in pools and hot tub users [60,61], include ear canal epithelium disruptions, such as abrasion (scratching, clearing ear canals), maceration, psoriasis, or eczema [60,61]. The likelihood of *P*. *aeruginosa* catheter-related urinary tract infections is increased following damage to the epithelium structure during catheter insertion or manipulation [62]. For respiratory infections, the significance of epithelial breaches is also observed, in addition to biofilm formation and impaired mucociliary clearance. HAE lesions are described in CF and COPD patients, but also in those undergoing intubation or mechanical ventilation [13,63–65]. (See below.)

#### 2) HAE breach allows *P*. *aeruginosa* access to the basolateral part of the epithelium

Epithelial breaches expose the basolateral part of the epithelium (i.e., the basolateral membrane of HAE cells and/or the basement membrane), which is normally inaccessible in healthy epithelium owing to intact tight junctions [34]. Access to the basolateral part is the main element of *P*. *aeruginosa* interaction and is crucial for initiating *P*. *aeruginosa* respiratory infections.

Many studies demonstrated that *P*. *aeruginosa* poorly interacts with an intact, healthy, and well-polarized HAE. Conversely, numerous reports demonstrated preferential interactions with injured or repairing epithelia, or with nonpolarized cells in the basolateral part of HAE. The first such report showed that in rat tracheal surface, *P*. *aeruginosa* interacts in vivo more easily with brush-injured sites than with those that are not injured [66]. Subsequent studies have also shown infrequent adhesion to the normal epithelium but increased adhesion to areas of epithelial damage and the basement membrane in airway culture models [9,67]. Lee and colleagues confirmed the crucial role of epithelial tight junctions in interaction with *P*. *aeruginosa*. They showed that bacterial adhesion and toxic effects are more frequently found near the free edges of epithelial wounds, where the basolateral cell plasma membrane is accessible [68]. HAE with intact tight junctions is entirely resistant to *P*. *aeruginosa*-induced cell apoptosis, as opposed to nonjunctional epithelia [69]. Although the mucociliary clearance decreases bacterial access to the epithelium, the Puchelle team reported that *P*. *aeruginosa* is unable to adhere to normal and uninjured cells because the functional tight junctions play a key role in masking the basolateral main receptors for bacterial attachment (see below). They described the preferential sites of interaction with *P*. *aeruginosa* on the exposed basolateral plasma membranes, the denuded basement membrane after injury, as well as the flattened migrating and spreading cells during repair [11,14,15,70,71]. Other studies confirmed that basolateral plasma membranes exposed after injury are more likely to bind *P*. *aeruginosa* and that the level of the polarity of the cells is also an important factor, given that nonpolarized migrating cells or incompletely polarized cells are more susceptible to *P*. *aeruginosa* binding and intoxication [72,73].

#### 3) Main settings wherein *P*. *aeruginosa* could exploit opportunities to gain access to the basolateral part of HAE

**Fig 2:**
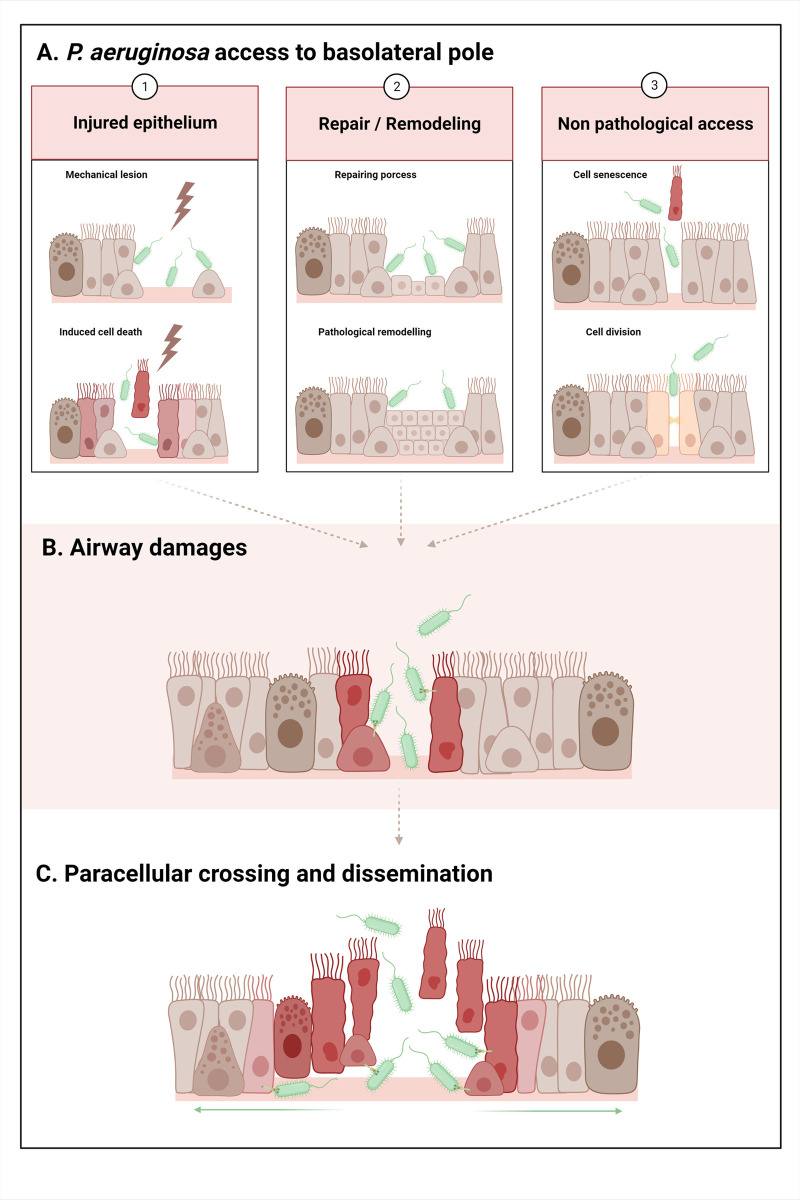
*P*. *aeruginosa* basolateral interaction and progression in the airway epithelium. **A.** In the first step of infection, *P. aeruginosa* gains access to the basolateral part, exploiting various opportunities: **1.** Injured epithelium: **a.** Mechanical lesion: removal of HAE cells and denudation of the basement membrane. Ex: endotracheal tube in VAP. **b.** Induced epithelial cell death: dying cells undergo retraction, tight junction disruption, and detachment from the adjacent cells and the basement membrane. The injury could be caused by pathogens (viruses, bacteria, etc.), chemical injury (pollutants, toxic compounds, etc.), or excessive inflammatory processes (excess of cytokines, proteases, oxidant stress, etc.). Ex: excess inflammation injuring HAE in CF and COPD. **2.** Repair of the epithelium after injury/epithelium remodeling: **a.** After an injury, HAE undergoes a repair process: basal cell dedifferentiation, spread and migration, transitional squamous metaplasia or basal/mucous hyperplasia, and progressive differentiation. Cells display low differentiation levels, low polarity, and no functional tight junctions. Ex: repair in CF and COPD. **b.** Chronic and pathologically remodeled epithelium: squamous and goblet cells metaplasia, hyperplasia of surface goblet and basal cells. Cells display low differentiation levels, low polarity, and no functional tight junctions. Ex: remodeled epithelia in CF and COPD. **3.** Nonpathological access in differentiated epithelium: transient disruption of tight junctions during: **a.** Extrusion of a senescent cell. **b.** Cell division. **B.** *P. aeruginosa* virulence factors induce airway damage, notably by T3SS toxin injection on the basolateral membranes of the cells. T3SS effectors (ExoS, ExoT, and ExoU) induce cell retraction and death, facilitating the subsequent access of bacteria to the adjacent or underlying cells and the basement membrane. **C.** Bacteria cross the epithelium by this paracellular route, gain access to the basal part, and progressively propagate radially through the epithelium to disseminate, using pili and flagella. Created with BioRender.com.

**a) Injured epithelium.** Following an injury to the epithelium, the basolateral part becomes accessible to *P*. *aeruginosa* because the basolateral membrane of adjacent cells and basement membrane are exposed (Fig 2A and left panel).

First, this can result from endotracheal tube insertion and manipulation that, not infrequently, induce mechanical damage in ventilator-associated pneumonia (VAP). The endotracheal tube can cause scratching and abrasion of the HAE, removing fragments of the epithelium and leading to denudation of the basement membrane [13,63,74]. In addition, biofilm formation on the plastic surface and mechanical impairment of the mucociliary clearance by the tube facilitate infection [63].

Second, this can result from induced HAE cell death. Dying cells undergo retraction and tight junction disruptions, leading to subsequent detachment from the adjacent cells and the basement membrane. The causes of cell injury are extremely diverse and can be induced by pathogens (viruses, bacteria, etc.), chemical injury (pollutants, toxic substances, allergens, etc.), or hyperactive inflammation (excess of cytokines, proteases, oxidant stress, etc.) [14,35]. Injuries could be part of a potentially vicious circle since HAE injury by itself will attract immune cells. For instance, degranulation of neutrophils contributes to inflammation and tissue damage [19,25,75,76], which could increase the number of breaches and thereby provide an opportunity for *P*. *aeruginosa* to reach the basolateral part of HAE. But, a fine-tuned homeostasis has been described in peritoneal serosa, which is likely transposable to other tissues including the lung tissue. Indeed, Uderhardt and colleagues reported that such homeostasis is maintained to prevent the excess of deleterious inflammation in the case of microlesion. Resident tissue macrophages sense rapidly the death of individual cells and sequester the damage owing to extending membrane processes (pseudopods), thus preventing neutrophil activation [77]. In case of too many lesions, such as exemplified below for CF and COPD, the cloaking by resident tissue macrophages is overwhelmed and would not prevent neutrophil-driven inflammation and subsequent tissue damage.

In CF disease, different factors contribute to *P*. *aeruginosa* infections. The most common and well-accepted paradigm is the “low volume hypothesis” stating that in the absence of a functional chloride channel CFTR, the airway mucus is dehydrated, hyperviscous, and thickened, thereby compromising the mucociliary clearance [78,79]. The mucus stagnates in the airways, and this aberrant accumulation of mucus provides a nidus for colonization and recurrent infections by opportunistic pathogens, like *P*. *aeruginosa* [78]. As CF patients become chronically infected, *P*. *aeruginosa* adapts to the specific lung environment by genotypic and phenotypic variations, such as loss of virulence and/or increased resistance to antimicrobials and host immunity. *P*. *aeruginosa* usually grows as a biofilm on host tissues/epithelial surfaces during these chronic infections [78,80]. Bacterial multiplication, following their entrapment within the mucus, induces a vigorous inflammatory response. Moreover, the deficit in the immune response due to CFTR dysfunction leads to persistent and hyperactive immunological stimulation, resulting in chronic lung inflammation [18,19,78]. For instance, the decreased CFTR functionality reduces bicarbonate secretion in the airways, then decreases the pH of the mucus in CF patients. It leads to the inactivation of cationic antimicrobial peptides secreted by the host and to mucus tethering and impaired mucus detachment from the lung epithelium [78]. Activated neutrophils and macrophages accumulated in the airways, and these cells release multiples products such as inflammatory cytokines (tumor necrosis factor (TNF)-α, interleukin (IL)-1, IL-8), reactive oxygen species, or protease (like neutrophil elastase), all contributing to the HAE destruction [19,25,75,76]. This excessive and ineffective inflammatory response, probably exacerbated by bacterial toxins, leads to the inadvertent destruction and damage of the HAE [14,18,19,25]. Various injuries to the HAE have been previously described in CF airways, including microlesions, shedding areas (few basal cells are still present or even total denudation of the basement membrane), epithelial sloughing and disorganization of the epithelial tight junctions, etc. [14,39,64,81,82].

Besides the mucociliary clearance elevator, the mucus composition could also play a role in bacterial virulence in the lung. In healthy epithelium, the mucus protects against pathogens’ epithelial adhesion and cytotoxicity and appears to play an important role in suppressing bacterial virulence. It was shown that mucins prevented *P*. *aeruginosa* aggregation and adhesion to the underlying surface and that mucins triggered the dispersal of *P*. *aeruginosa* biofilm[83,84]. Mucins significantly enhanced twitching motility and decreased biofilm formation [85]. It was also described that mucins attenuated the virulence of *P*. *aeruginosa*, decreasing many virulence genes (such as type 3 and 6 secretion systems (T3SS/T6SS), siderophores, and quorum sensing), disintegrating biofilms, and reducing cytotoxicity on human epithelium colorectal cells and burn infections in a porcine model [86]. When mucus structure and/or properties are compromised, the mucus’ protective abilities may be significantly decreased. It is exemplified in CF with a defect of mucin expression and mucus production associated with more susceptibilities to *P*. *aeruginosa* infection than in the healthy lung [50].

In COPD patients, the chronic and excessive inflammation due to the causative inhaled irritants (for instance, cigarette smoke) leads to an increased number of neutrophils and macrophages in the lungs, as well as to the activation of airway epithelial cells and mucus hypersecretion. Emphysema and luminal occlusion by aberrant mucus and inflammatory exudate is commonly observed [87]. A reduced mucociliary clearance is also described in COPD [88]. Cells produce pro-inflammatory cytokines, such as TNF-α, IL-1β, IL-6, and IL-8, and release elastase. Combined with the products of oxidative stress, it contributes to the HAE breakdown [87,88]. Microbial colonization of the respiratory tract is often observed, which can be associated with acute exacerbation, enhancing the host inflammatory response [7]. This combined pathogen and host actions enhance HAE injuries [7]. Particularly, in COPD, the major alteration of the lung is parenchymal destruction [65]. Coinfections with viruses and bacteria have been described to cause more severe functional lung impairments in COPD patients [89]. Viruses, notably the respiratory syncytial virus, alter the expression of receptor molecules on respiratory epithelial cells and provoke cell death. In turn, an increase in the adhesion and invasion by *P*. *aeruginosa* leads to coinfections or superinfections [7,90].

**b) Repair of the epithelium after injury/epithelium remodeling.** To regenerate and restore its function following injury, the HAE has to repair itself. During this process, the surviving basal cells that are adjacent to the wound edge, spread, and migrate to cover the denuded basement membrane [35,91]. Basal cells then proliferate, forming a transitional squamous metaplasia [37]. Cells undergo a differentiation process, progressively regenerating their polarity and reform junctions, resulting in a complete and functional pseudostratified mucociliary epithelium. During this repairing process, the exposed cells display low or no differentiation levels, low or no polarity, no functional tight junctions, and expressed alternative receptors, favoring interactions with *P*. *aeruginosa* [14,15,35,43,70,72] (see “Adhesion” section and Table 1). As for injuries described above, for CF and COPD, *P*. *aeruginosa* interactions with repairing HAE are facilitated (Fig 2A and middle panel).

In CF and COPD, chronic and pathological remodeling, such as squamous metaplasia or hyperplasia of goblet and basal cells, is typically observed [34,38–42,65,82]. As in the repairing process, cells in remodeling epithelia show reduced differentiation, low polarity, absence of functional tight junctions, and expression of alternative receptors, again enhancing their potential interaction with *P*. *aeruginosa* [14,15,70] (see “Adhesion” section and Table 1).

**c) Access in nonpathological conditions** Bacteria can also gain access to the basolateral part of the HAE in differentiated epithelium without any injury or repair process, due to the normal epithelial renewal. Some studies have shown that after a transient disruption of the epithelial junctions, either following extrusion of a senescent cell or cell division during multiplication, the basolateral part becomes exposed and *P*. *aeruginosa* can interact with the HAE [92–94]. As previously described for the pathogenic bacteria *Listeria monocytogenes* in the MDCK cell line [95], Heiniger and colleagues described that *P*. *aeruginosa* adhesion to the apical membrane of ciliated cells from primary differentiated human epithelium is very low (see “Adhesion” section). They also showed that the interaction with the cells occurs after a transient disruption of the epithelial barrier during the extrusion of a senescent cell. The basolateral membrane of the ciliated cells is then exposed, and the bacteria can easily interact with it [94]. Disruptions of tight junctions occur during cell divisions or cell senescence and are both natural phenomena ensuring epithelial homeostasis [92,93]. In the MDCK cell line model, Golovkine and colleagues showed that very soon after HAE infection (approximately 3 hours), *P*. *aeruginosa* takes advantage of these brief and transient ruptures of the epithelial barrier to gain access to the basolateral part. In this model, cell deaths were not caused yet by *P*. *aeruginosa* but were natural/physiological deaths [93] (Fig 2A and right panel).

The clinical impact of the interaction of *P*. *aeruginosa* to the basolateral part in vivo in healthy humans might be low and insufficient to develop an infection, as *P*. *aeruginosa* is rarely described in community-acquired pneumonia [96]. The functional mucociliary clearance and competent immune defenses are likely to clear most of the *P*. *aeruginosa* present in the airways of healthy individuals. Moreover, airway cell divisions/senescence are infrequent, decreasing the probability of encountering *P*. *aeruginosa* to initiate infections in healthy individuals. Indeed, (i) the cell turnover rate of the HAE self-renewal is one of the lowest compared to other epithelia, more than 100 days for HAE [97,98] versus 3 to 5 days for intestinal epithelium, 10 days for corneal epithelium, or 20 days for cutaneous epithelium [99–102]; (ii) Golovkine and colleagues described that the opportune interaction of *P*. *aeruginosa* is not systematic in their model but is rather a rare event since many sites of cell divisions/deaths were not exploited by the bacteria [93]; (iii) the cloaking by resident tissue macrophages prevents excess tissue damages and preserves tissue homeostasis [77]; and (iv) the few cases of *P*. *aeruginosa* community-acquired pneumonia, described in healthy individuals, were often associated with prolonged or repeated exposures to contaminated whirlpools or hot tubs aerosols [61,103–105]. The number of *P*. *aeruginosa* observed in these contaminated waters, wherein the bacteria are proliferating, was high (up to 10^5^ colony-forming units (CFU)/mL) [61,105–107]. We can speculate that the high infectious inoculum of *P*. *aeruginosa* can counter the strong barrier effect by increasing the probability of encountering the rare physiological breach event, thereby explaining the development of infection in a healthy individual.

Either way, it is noteworthy that the studies describing *P*. *aeruginosa* interaction with intact airway epithelium in ex vivo culture models used very high bacterial inocula (from 10^6^ to 10^8^ CFU [72,94,108–112]) or high concentration of purified virulence factors (such as elastase [113–117], LPS [118], rhamnolipids [119,120], and 3OC12-HSL [121–123]). Although both are related to the bacterial load commonly detected in clinical samples [124,125], they correspond to thresholds commonly seen in clinical microbiology laboratories at the time of airway infection diagnosis. Considering the bacterial proliferation in the infected airways, they are likely far higher than the inoculum at the initiation of the epithelial breach. Additional studies are needed to address the relevance of the infectious inoculum in vivo, when *P*. *aeruginosa* pulmonary infections occur in CF, COPD, or VAP.

### The steps of progression of *P*. *aeruginosa* during airway infection

#### 1) Adhesion of *P*. *aeruginosa* to the airways

**Fig 3:**
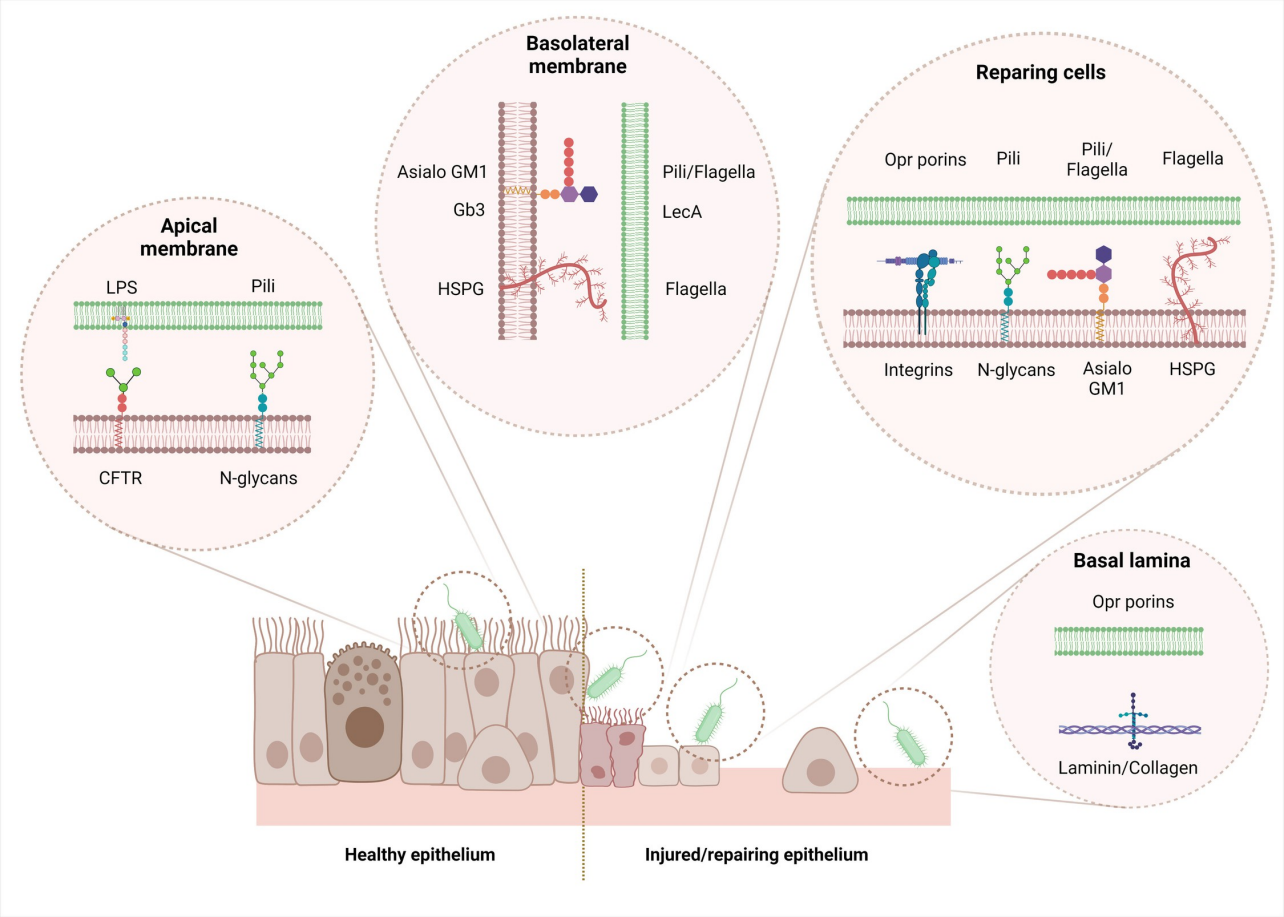
Airway receptors of *P*. *aeruginosa*. Green membrane: *P*. *aeruginosa*; brown membrane: host cells. Created with BioRender.com.

Respiratory infections by *P*. *aeruginosa* are usually initiated by the adhesion of the bacteria to the HAE cells or the basement membrane. Appendices of motility and adherence, pili, and flagella are virulence factors present on the bacterial surface, mainly responsible for movement and attachment to the cells [109]. Utilizing different mechanisms of motility (swarming, swimming, and twitching), the bacteria sense the optimal surface for initiating cell surface contact and properly attached to the surface [126]. Several molecules, present on the host epithelial cell and extracellular matrix components and containing long carbon chains, bind the different adhesins of *P*. *aeruginosa* (Fig 3 and Table 1).

**Table 1:** *P*. *aeruginosa* airway receptors.

	Airway receptors	Type of cells used to evidence airway receptors	Location in healthy airways	Pathological epithelia	PA adhesin	References
**Glycosphingolipids (Lipid rafts components)**	**Asialo GM1 (GalNAcβ1-4Gal disaccharides)**	Primary human nasal epithelial cells (differentiated and not differentiated)	Basal cell membrane	Specific apical membrane expression by regenerating epithelium or CF epithelium	- Type IV pili (C-terminal part) - Flagella	[16,127–131]
	**Globotriaosylceramide Gb3 (CD77, αGal–βGal–βGlc–Cer saccharides)**	Cell lines (H1299 kc, A549)	Unknown	-	LecA	**[132–137]**
**N-glycoproteins**	**N-glycans**	Cell lines (Calu 3) Primary bronchial cell	Apical membrane mainly (expressed on basal cell membrane, but few interactions with PA)	Enhanced expression in damaged epithelium	Type IV pili	[109,138,139]*
	**CFTR**	Cell lines (CFT-1, CFBE41o-, A549, WI-38) Primary human bronchial epithelial cells (differentiated)	Apical membrane	Decreased expression in dedifferentiated and remodeled epithelium	LPS	**[140–145]**
**Integrins**	**α5β1 integrin–Fibronectin**	Cell lines (16HBE) Primary nasal cells (differentiated)	Absent in normal airways	Apically exposed and overexpressed in repairing epithelium	OprQ^a^	**[70,71,146–148]**
	**αvβ5 integrin–Vitronectin**	Bronchial tissue	Unknown	Increased expression in repairing epithelium or inflamed epithelium	OprD	**[149–152]**
**Proteoglycans**	**HSPG (Heparan sulfate proteoglycans) (HS chain)**	Cell lines (Calu 3, 16HBE) Primary nasal cell	Basal cell membrane mainly	Apically increased during epithelial injury and dedifferentiation	Flagella	[109,138,153,154]*
**ECM**	**Laminin Type I and IV collagens**	Not applicable	Basement membrane (ECM beneath the epithelium)	Accessible only in injured epithelium	OprD, OprG, EstA, PA3923^b^	**[14,155,156]**

CFTR, cystic fibrosis transmembrane regulator; ECM, extracellular matrix; LPS, lipopolysaccharides; OMP, outer membrane protein; PA, *P*. *aeruginosa*.

^a^Roger and colleagues described a 50-kDa OMP, probably corresponding to the OprQ porin described later by Arhin and colleagues.

^b^de Bentzmann and colleagues described 60 kDa OMPs, probably corresponding to the OprD, OprG, EstA, PA3923a porin described later by Paulsson and colleagues.

*References [139,154], cited for the localization of the airway receptor, were performed in another context than *P*. *aeruginosa* infection.

It is noteworthy that some of these receptors, listed in Table 1, are expressed at different locations on the epithelium, depending on specific situations. At the apical side of healthy airway epithelium, very few receptors are present, which strongly limit interaction with *P*. *aeruginosa*. For instance, integrins or N-glycans are hardly or not at all present at the apical part of healthy epithelium, but they are overexpressed during the repair process specifically [138]. Asialo GM1 and HSPG are mainly expressed at the basolateral part but are specifically recovered at the apical part in regenerating or CF epithelia [16,128,138]. Migrating cells actively synthesize cellular fibronectin/vitronectin, and their receptors, the integrins, are up-regulated and apically exposed during migration in the repairing process [70,148,151]. As fully detailed in Part I, the adhesion of *P*. *aeruginosa* to receptors at the basolateral part of the epithelium can only occur in case of disruption or lack of tight junctions or when cells lose their polarization, which is mainly observed within injured or repairing/remodeled epithelium [9,14–16,67,68,94].

Pili-mediated adhesion, combined with their retractive ability, creates close contact between bacteria and host cells and induces the action of various export systems. In particular, the T3SS is an important contact-dependent virulence factor that injects toxins through the membrane of the host cell [157,158]. Pili mechanical retraction induces the chemosensory system phosphorelay (Chp), leading to stimulation of adenylate cyclase CyaB and up-regulating the signaling molecule adenosine-cyclic monophosphate (cAMP) production, which allosterically activates the virulence factor regulator (Vfr)-dependent virulence system [158,159]. Vfr regulates multiple virulence factors in *P*. *aeruginosa*, including the T3SS, but also the T2SS and the quorum sensing [160,161].

#### 2) Airway damage induced by *P*. *aeruginosa*

*P*. *aeruginosa* virulence factors involved in respiratory infections

*P*. *aeruginosa* is an environmental bacterium adapted to the soil and aqueous habitat. It is an opportunistic pathogen found mainly in immunocompromised patients. Human airway pathogenicity of *P*. *aeruginosa* is explained by the production of many virulence factors, displaying varied and complementary properties [162,163]. Some factors are involved in motility and adhesion, persistence in the host, iron uptake, or resistance to oxidative stress. The toxins are mostly involved in the destruction of the host cells [163].

**Fig 4:**
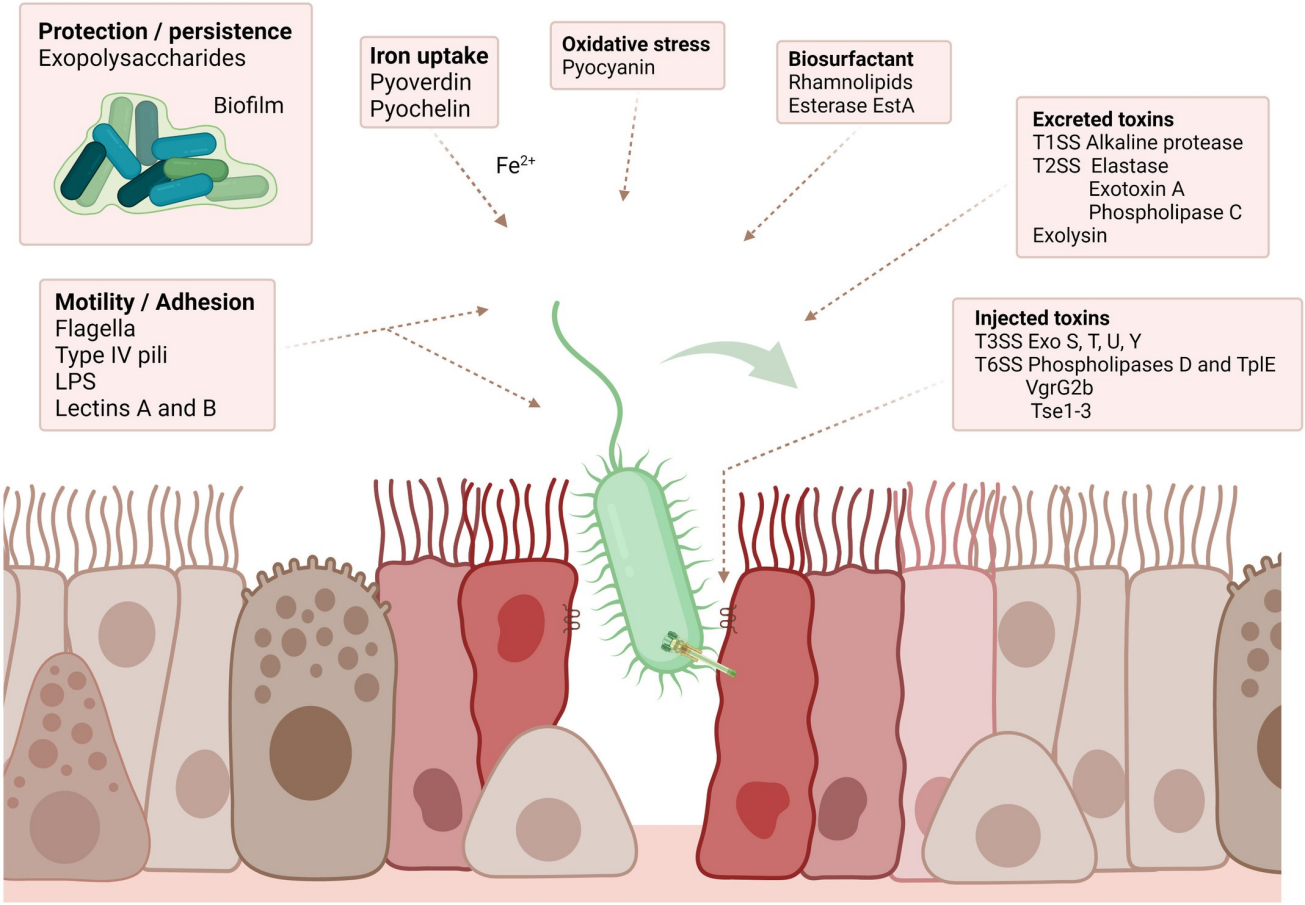
*aeruginosa* main virulence factors in respiratory infections. ***P*.** Created with BioRender.com.

The main virulence factors of *P*. *aeruginosa* involved in respiratory infections are illustrated in Fig 4 and their functions are detailed in Table 2.

**Table 2:** Virulence factors of *P*. *aeruginosa* in respiratory infections.

Class	Virulence factor	Gene(s)	General function(s)	HAE-specific function(s)	Type of cells used to evidence HAE-specific function(s)	Molecular host target	SS	Reference
**Motility/Adhesion**	Flagella	*fliC*	Swimming and swarming motility Host cell adhesion and internalization	Motility and basal adhesion to HAE cells HAE infection progression	Primary nasal cells, cell lines (Calu 3, 1HAEo-), mice pneumonia	Heparan sulfate, proteoglycans, AsialoGM1	-	[127,130,138,172]
	Type IV pili	*pilA*	Twitching motility Host cell adhesion, biofilm formation	Motility and apical adhesion to HAE cells Allow T3SS injection. HAE infection progression	Cell lines (Calu 3)	N-glycans	-	[138,173,174]
	LPS	*waa*, *wzy*, *wzz*, *wbp*, *wzx*	Resistance to serum killing and phagocytosis, inflammatory response Binding to CFTR Cell junctions’ disruptions and apoptosis	Apical adhesion to HAE cells Disruption of HAE tight junctions HAE cell apoptosis	Cell lines (A549, NCL-H292, Beas-2b)	CFTR, Tight junctions (ZO-1)	-	[54,118,175–177]
	Lectin A Lectin B	*lecA* *lecB*	Host cell adhesion: binding to galactose (LecA) and fucose (LecB) Internalization (LecA) Cytotoxicity (LecA and LecB)	Apical and basal adhesion to HAE cells HAE cell internalization HAE cell cytotoxicity	Primary nasal cells, cell lines (H1299, A549), mice pneumonia	Globotriaosylceramide Gb3 (CD77)	-	[132,134,178]
**Protection/Persistence**	Exopolysaccharides (Alginate, Psl, Pel)	*alg*, *muc*, *psl*, *pel*	Biofilm main component: polymeric matrix Persistence, bacteria protection (from IS and antibiotics)	Biofilm persistence in chronic HAE infection	-	-	-	[167,179–181]
**Iron uptake**	Siderophores: - Pyoverdin - Pyochelin	*pvd* *pch*	Iron siderophore, green pigment (pyoverdine) Cytotoxicity (ROS production)	-	-	DNA, lipid membrane, proteins	-	[182–185]
	Heme uptake systems: *- Pseudomonas* heme uptake - Heme assimilation systems	*phu* *has*	Extracellular heme acquisition (uptake in the cytoplasm)	-	-	-	-	[185–187]
**Oxidative stress**	Pyocyanin	*phz*	Cytotoxicity (ROS production: O_2_^−^, H_2_O_2_) IS regulation (apoptosis), blue-green pigment	Inhibition of ciliated HAE function	Primary sheep cells	DNA, lipid membrane, proteins	-	[188–190]
**Biosurfactant**	Rhamnolipid	*rhl*	Amphiphilic: detergent and solubilizing properties Disruption cell junctions IS regulation (neutrophils lysis) Biofilm formation, motility	Solubilization of airway surfactant Disruption of HAE tight junctions	Primary nasal cells	Tight junctions (ZO-1, occludin, JAM-A)	-	[120,191–193]
	Esterase EstA	*estA*	Autotransporter enzyme: Hydrolyze glycerol esters Rhamnolipids production	Disruption of HAE tight junctions	See Rhamnolipid	See Rhamnolipid	-	[194]
**Excreted toxins**	Alkaline protease	*aprA*	Protease activity: IS inactivation (antibodies, neutrophils, complement, cytokines) and laminin	Degradation of HAE extracellular matrix: (basement membrane: laminin)	-	Laminin	T1SS	[195,196]
	Elastase	*lasB*, *(lasA)*	Protease activity: elastin, collagen, transferrin, antibodies, complement → Tissue damage Disruption of cell junctions	Degradation of HAE extracellular matrix (basement membrane and alveolar septum: elastin and collagen) Disruption of HAE tight junctions	Primary nasal cells, cell lines (Calu 3)	Elastin, Collagen, tight junction (ZO-1, claudin, occludin)	T2SS	[113,114,117,197]
	Exotoxin A	*toxA*	Host cells protein synthesis inhibition: eEF2 inhibition by ADP-ribosylation → Cell death	HAE cell death	Cell lines (CuFi-1)	eEF2	T2SS	[198,199]
	Phospholipase C	*plcH*	Hydrolysis of phospholipids (erythrocyte and leukocyte cytolysis), surfactant degradation	Pulmonary surfactant degradation	-	Phosphatidylcholine, Sphingomyelin	T2SS	[200–203]
	Exolysin	*exlA*	Pore-forming toxin: cell membrane disruptions (erythrocytes, leukocytes, epithelial cells)	HAE cell death and retraction	Cell lines (A549), mice pneumonia	Phophoslipid bilayers	-	[108,204,205]
	CFTR inhibitory factor	*cif*	-	TAP-1 mediated-MHC class 1 antigen presentation and CFTR-mediated chloride secretion inhibition	Cell lines (CFBE41o-, A549)	CFTR and TAP-1	OMV	[206,207]
**Injected toxins**	Exo S (exoenzyme S)	*exoS*	GTPase-activating protein activity and ADP ribosyltransferase activity: inhibition of several host cell functions (cell apoptosis, cell division and cell migration inhibitions, junctions and actin cytoskeleton disruptions)	HAE cell death and retraction	Mice pneumonia	Rho family of GTPases, Ras superfamily GTPases	T3SS	[163,208,209]
	Exo T (exoenzyme T)	*exoT*						
	Exo U (exoenzyme U)	*exoU*	Phospholipase A2 activity: membrane phospholipids hydrolysis (rapid cell necrosis)	Rapid HAE cell death and retraction	Mice pneumonia, cell lines (Beas-2b)	Phospholipids	T3SS	[163,208,210]
	Exo Y (exoenzyme Y)	*exoY*	Adenylate cyclase activity: actin cytoskeleton disruptions	HAE cell death and retraction	Mice pneumonia, cell lines (PMVECR1)	Tau protein (microtubule)	T3SS	[211–214]
	Phospholipase D	*pldA*, *pldB*	- Host cell internalization - Bacterial competition	HAE cell internalization	Cell lines (Calu 3)	Akt kinase	H2 (*pldA*) H3 (*pldB*) T6SS	[110,215,216]
	VgrG2b	*vgrG*	- Host cell internalization - Bacterial competition	HAE cell internalization	Cell lines (Calu 3	γ-tubulin ring complex (γTuRCn microtubule component)	H2 T6SS	[110,217]
	Phospholipase TplE	*TplE*	- Host cells endoplasmic reticulum disruption - Bacterial competition	-	-	Endoplasmic reticulum apparatus	H2 T6SS	[218]
	Tse1-3	*Tse1-3*	Bacterial competition (peptidoglycan degradation)	-	-	-	H1 T6SS	[215]
**Short RNA**	sRNA52320	*-*	-	Decrease of IL-8 secretion	Bronchial primary cells	Kinases of the LPS-stimulated MAPK pathway	OMV	[219]
**Global regulatory system**	Quorum sensing	*las*, *rhl*, *pqs*, *iqs*	Autoinducer peptides (for instance, homoserines lactones 3O–C12–HS) detect critical density → activation of 4 regulation systems: impact on virulence factors production and biofilm formation	Disruption of tight junctions^a^	-	Tight junctions (ZO-1, ZO-3, JAM-A, occludin)	-	[122,123,164,165,220]

^a^Disruption of cells’ tight junction described in the intestinal epithelial model (Caco2 cells), not yet in HAE.

CFTR, cystic fibrosis transmembrane regulator; eEF2, eukaryotic elongation factor 2; HAE, human airway epithelium; H_2_O_2_, hydrogen peroxide; LPS, lipopolysaccharide; MHC, major histocompatibility complex; OMV, outer membrane vesicle; O^2^, superoxide; IS, immune system; ROS, reactive oxygen species; SS, secretion system; TAP, transporter associated with antigen processing; TxSS, type x secretion system.

Most of these virulence factors are not synthesized constitutively but are regulated depending on the needs of the bacteria in different types of settings and collaterally during infection. The production is fine-tuned by quorum sensing, which is a communication system based on the detection of bacterial density in the environment [164]. The communication is mediated by the secretion of small signaling molecules, such as homoserines lactones or quinolones, used as autoinducer messengers. Each bacterium produces these messengers, thereby their concentration depends on the growing bacterial population [164]. When the critical threshold is reached (i.e., *quorum*), four regulatory systems are activated to coordinate the response of the bacterial population: Las, Rhl, PQS, and IQS systems [164–167]. Around 10% of the *P*. *aeruginosa* genome is estimated to be regulated by the quorum sensing system [168].

In some conditions, and notably in CF disease, *P*. *aeruginosa* is known to form structured aggregates known as biofilms. After adhesion to a surface, the bacteria congregate in highly organized communities, surrounded by an extracellular matrix composed of exopolysaccharides (alginate, PEL, and PSL), extracellular DNA, lipids, and proteins [169]. This matrix, representing between 50% and 90% of the total volume of the biofilm, strengthens its structure and protects bacteria from effectors of the host’s immune response (such as phagocytosis or antibody actions) and antibiotics, making their eradication very difficult [18,167,169]. In addition, bacteria within the biofilm are exposed to oxygen gradients and nutrient limitations leading to modification of bacterial metabolism. One clinically relevant phenotype that occurred in such a situation is the small colony variant phenotype, which is characterized by slow growth and a loss of cytotoxicity [25]. Biofilm formation is regulated by the quorum sensing system [18,165]. The development of the mucoid phenotype by overproduction of alginate found in biofilms is a strategy for the bacteria to survive in a hostile environment and is mainly observed in *P*. *aeruginosa* chronic respiratory infections (such as in CF patients) and biomaterial-associated infections (endotracheal tubes, urinary catheters, etc.) [25,170]. The inert surfaces of the biomaterial are used as a base that facilitates the development of biofilms [18,171].

During the progression of chronic *P*. *aeruginosa* infection in CF patients, phenotypic and genotypic changes occur in *P*. *aeruginosa* to adapt to the specific mucus-plugging environment. Strains established in this long-term colonization show less inflammation and less cytotoxicity against HAE than the first strains years earlier in the same patient [18]. A switch from acute to chronic phenotype occurred. It has been described to be characterized by down-regulation of some virulence factors (motility with loss of flagellum and pili, protease, rhamnolipids, pigments, …), increased biofilm formation, the apparition of mucoid or small colony variant phenotype, change in LPS, and alteration of quorum sensing system [18,78].

From the first stages of *P*. *aeruginosa* interaction with the airway epithelium, the first line of defense of the innate immune system in the lung recognizes *P*. *aeruginosa* components and virulence factors, activating an immune reaction to prevent or resolve the infection. Various immunity actors are then recruited, and inflammation processes are observed [19].

First, specific *P*. *aeruginosa* patterns are recognized thanks to pattern recognition receptors (PRRs) [19,221]. Three types are described: (i) Secreted PRR, such as Complement C1q, collectins, and surfactant proteins SP-A and D, which bind *P*. *aeruginosa* cell wall and mark it for clearance by macrophages and neutrophils. (ii) Transmembrane PRR, the most important actors, represented by the Toll-like receptors (TLRs) present on immune and epithelial cell membranes. They recognize different components of *P*. *aeruginosa* call the PAMPs (pathogen-associated molecular patterns). For example, TLR2 recognizes the pili, TLR4 and CFTR the LPS, and TLR5 the flagella. (iii) Cytosolic PRRs, such as TLR9 and NOD-1, can recognize bacterial DNA or cell wall components of the internalized/phagocytosed bacteria.

Various cells coordinate efforts to produce an appropriate innate immune response [18,19,78]. Epithelial cells of the HAE play an important role of alert of *P*. *aeruginosa* presence. After TLR-mediated activation, they produce a large panel of pro-inflammatory cytokines (TNF-α, IL-1β, IL-2, Il-6, …) and the chemokine IL-8, which initiate inflammation and recruit other more specialized immune cells such as neutrophils. They also produce antimicrobial peptides (defensin, cathelicidin LL-37) and reactive oxygen species (radical hydroxide, hypothiocyanite), acting directly against *P*. *aeruginosa*. Neutrophils play a critical role in *P*. *aeruginosa* destruction and the development of the inflammatory response. Not present in the uninfected lung, they are recruited by attractive chemokines released by sentinel cells during infection, such as macrophages or epithelial cells, mediated by PRR signals. They phagocyte and kill the bacteria and they generate a great number of antibacterial molecules, such as reactive oxygen species (superoxide anion, hydrogen peroxide, radical hydroxide, nitric oxide, responsible for an oxidative burst), proteases (elastase), and antimicrobial peptides (defensin, cathelicidin LL-37), lactoferrin and lysozyme. Alveolar macrophages are the resident leukocyte of the lung, acting as the main sentinel for infectious agents’ detection. They recognize *P*. *aeruginosa* with their PRR, phagocytose it, and complete the immune cells activation of the HAE cells, through pro-inflammatory cytokines secretion.

After this review of all *P*. *aeruginosa* virulence factors involved in respiratory infection, we will focus on the most important damages described in airway infections, caused by the main virulence factors secreted by *P*. *aeruginosa*. Two types of degradation will be described: (i) HAE cell damages, wherein the key role of T3SS in host cell retraction and disruption of tight junctions facilitates the subsequent access of bacteria to the adjacent or underlying cells (ii) airway components degradation (basement membrane, surfactant), allowing the radial progression of *P*. *aeruginosa* in the epithelium (Fig 2B and 2C).

**HAE cells damages: The key role of the T3SS cytotoxic activity**

Among all the virulence factors produced by *P*. *aeruginosa*, the T3SS and its effectors are commonly considered the major determinant of virulence during infection [157]. This extensively studied secretion system plays a key role in the *P*. *aeruginosa* pathogenesis by injecting effectors that include toxins directly into the cytoplasm of the host cells including HAE cells [157]. *P*. *aeruginosa* strains that do not express T3SS are less virulent in human clinical infections and animal infection models [157,222,223].

T3SS-mediated delivery of the exoenzymes requires the adhesion of the bacteria to the host cell. As previously described, attachment via pili and their retraction activate signaling pathways, mediated by the Chp system, and cAMP/Vfr circuit, which lead to the transcription of virulence-associated genes, notably T3SS effectors [158,160,161]. Four well-known T3SS effectors have been described (exoenzymes ExoS, ExoT, ExoU, and ExoY), but not all are produced by every *P*. *aeruginosa* strain. ExoT and ExoY are found in nearly all strains, but ExoS and ExoU are mutually exclusive [224,225] and rarely found together in the same strain [226,227]. Two types of *P*. *aeruginosa* strains are therefore observed, with distinct pathogenesis phenotypes: (i) the “ExoU profile,” expressing ExoT, ExoU, and ExoY, is highly virulent, induces rapid cell death of the host cells, and shows a low rate of cell internalization; and (ii) the “ExoS profile,” expressing the ExoS, ExoT, and ExoY, causes slower death and exhibits a higher rate of internalization. The phenotypes associated with the expression of “ExoU profile” are mainly encountered in acute infections (bacteremia, ocular infections, acute pneumonia, etc.), while the “ExoS profile” is predominant in pulmonary chronic infections (in CF, COPD, etc.) [163,208,228–230].

ExoU, which is considered the most potent of type 3 secreted toxins, has a phospholipase A2 activity that induces host cell membrane disruption by phospholipids hydrolysis. It leads to rapid necrotic death of both epithelial and immune cells. ExoU was mainly described in severe and acute infections, especially respiratory infections [163]. ExoS and ExoT are bifunctional proteins both displaying two distinct enzymatic activities: an N-terminal GTPase-activating protein (GAP) activity and a C-terminal adenosine diphosphate ribosyl-transferase (ADPRT) activity. Inhibiting host cell GTPases, such as Rho-, Rac-, Ras-, Rap-, and Cdc42 GTPases, ExoS, and ExoT cause actin cytoskeleton depolymerization of the HAE cells. It eventually results in the detachment of HAE cells from the basement membrane due to cell retraction (disruption of the cell structure and subsequent tight junctions disruption), cell rounding and apoptosis [157,163,231].

**Fig 5:**
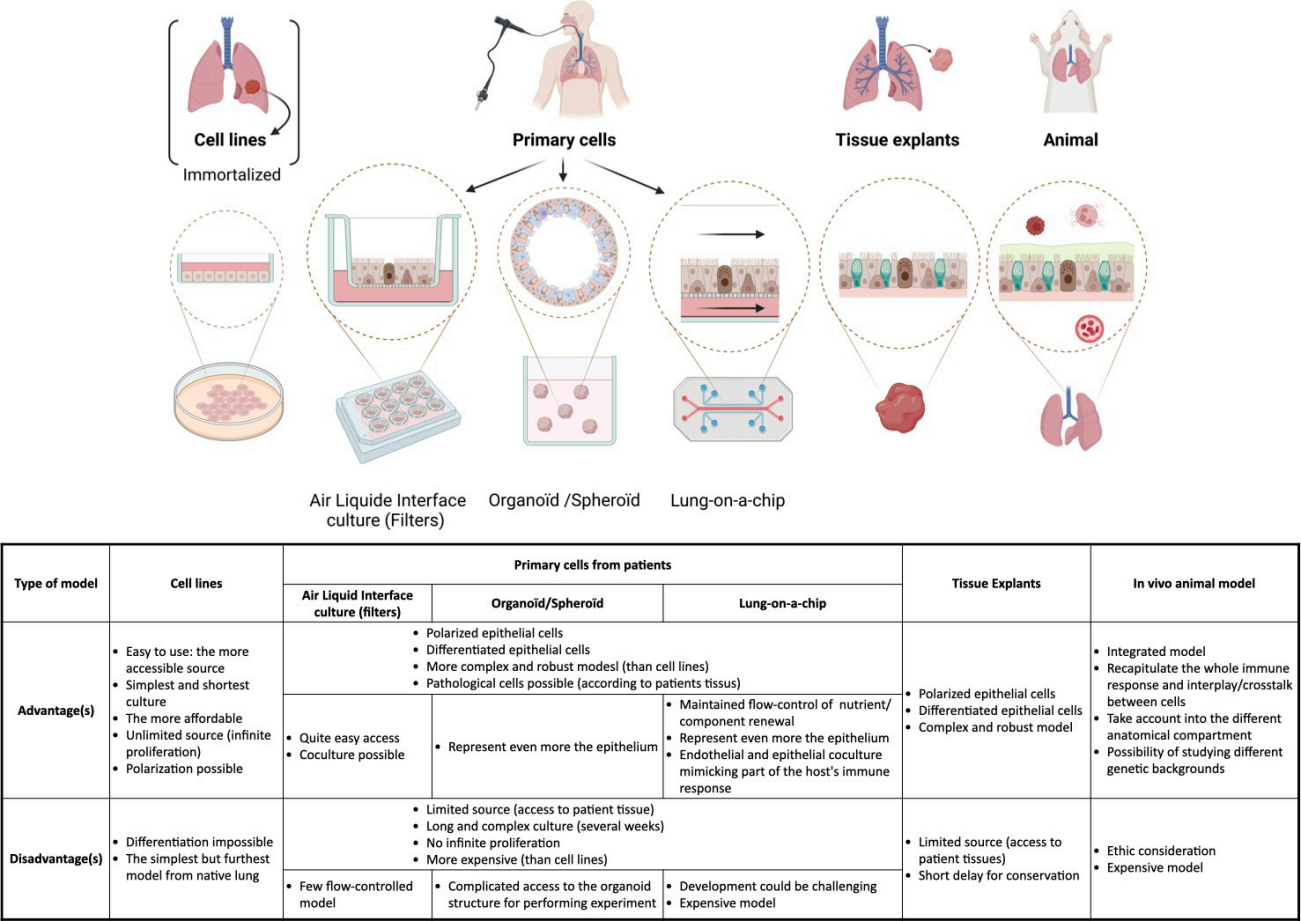
Models of airway epithelia. It is of the utmost importance to use relevant experimental models to assess the host–pathogen interactions driving *P*. *aeruginosa* infection, depending on the physiological relevance needed: Various models exist, from cell lines to primary cells and tissue explants/animal models, each with their advantages and disadvantages. All the models used in the references of the review are listed in S1 Table. Created with BioRender.com.

It is worth noting that the studies describing preferential T3SS cytotoxicity towards the basolateral part rather than the apical surface of HAE used mainly differentiated epithelium models, such as human or bovine primary airway epithelial cells cultured at the air–liquid interface (from 7 days to 6 weeks) [68,72,94] (Fig 5 and S1 Table). Such models likely mimic more in vivo conditions than immortalized cell lines [232].

Altogether, T3SS effectors play a key role in HAE damaging by facilitating the subsequent access of bacteria to the basolateral surface of other HAE cells nearby, allowing *P*. *aeruginosa* to break through the epithelium (Fig 2B and 2C).

Other secreted toxins or membrane constituents of *P*. *aeruginosa* have been shown to act complementarily to T3SS leading to enhanced cytotoxicity on HAE cells. For instance, the T2SS-secreted Exotoxin A catalyzes the ADP-ribosylation of its host target protein, the eukaryotic elongation factor 2 (eEF2), resulting in the inhibition of protein synthesis and ultimately leading to cell death [198,199]. The LecA and LecB binding proteins have also been shown to participate, at least in part, in a cytotoxic effect on HAE cells and to lung injury in a murine model [134]. The LPS can also induce apoptosis in lung endothelial and epithelial cells [177]. ExlA exolysin, a pore-forming toxin recently discovered in some strains of *P*. *aeruginosa* lacking T3SS, forms pores in the host cell membrane, leading to a massive entry of Ca^2+^ that in turn activates the ADAM10 metalloprotease [233]. The subsequent cleavage of the adherens junctions constituent E cadherin leads to HAE cell membrane disruption and death [108,205].

Some virulence factors affect the cells’ tight junctions and allow *P*. *aeruginosa* tissue progression. The proteolytic LasB elastase disrupts the tight junctions by acting on occludin, claudin, or ZO-1 proteins [113,114,116,117]. Rhamnolipids could also play a role thanks to their amphiphilic properties. They are incorporated into membranes and disrupt the architecture of tight junctions after several hours of infection in culture models [119,120,193]. In the same way, the LPS, a membrane component of *P*. *aeruginosa*, increases airway epithelium barrier paracellular permeability by disrupting the tight junctions, by affecting ZO-1 and ZO-2 [118,176]. Although studied only in an intestinal epithelium model, the homoserine lactone 3O–C12–HS of the *P*. *aeruginosa* quorum sensing has been shown to induce rupture of epithelium integrity [121]. It activates various cellular kinases implicated in tight junction’s functions (p38 or p42/44), leading to a modification of ZO-1, ZO-3, JAM-A and occludin production, as well as a reorganization of the actin cytoskeleton [121–123].

However, the inoculum of *P*. *aeruginosa* in the healthy epithelium is likely to be very low and the produced quantity of these factors might probably not be sufficient to break down alone intact tight junctions. Their impact is more likely delayed in *P*. *aeruginosa* epithelial invasion, after access to the basolateral part and bacterial proliferation in the infected airways, resulting in an inoculum far higher (commonly detected from 10^5^ to 10^7^ CFU/mL in respiratory clinical samples [124,125]. The role of these components might be then complementary to the action of T3SS-secreted exoenzymes in tissue progression.

**Degradation of other components of respiratory barriers**

In addition to the factors leading to the breakdown of the cells in respiratory epithelia, *P*. *aeruginosa* secretes several factors capable of destroying various components of the extra-epithelial components of the respiratory barriers, such as the basement membrane, the surfactant, and the mucins, which promote bacterial spread in the epithelium (Fig 2B and 2C).

*P*. *aeruginosa* produces proteases that play an important role in the degradation of the basement membrane and the mesenchyme. For instance, the LasB elastase, the most important protease of *P*. *aeruginosa*, degrades elastin and collagen and participates in tissue invasion and progression into the lung tissue [234]. The alkaline protease cleaves notably laminin, another important and biologically active component of the basement membrane [195].

Several other factors are involved in the degradation of the pulmonary surfactant: phospholipase C, which hydrolyses phospholipids [201,203,235], and the rhamnolipids, amphiphilic glycolipids having detergent and solubilizing properties [192].

It has been shown that *P*. *aeruginosa sdsA1* gene, encoding a secreted sulfatase, plays a central role in the degradation of mucin and that the *sdsA1* inhibition decreased the release of sulfate from mucin. *sdsA1* mutant showed a decreased mucin gel penetration and an attenuation of *P*. *aeruginosa* virulence in leukopenic mice intraperitoneally infected [236].

#### 3) *P*. *aeruginosa* internalization in HAE cells

Although *P*. *aeruginosa* is primarily an extracellular pathogen, internalization in nonphagocytic cells, such as airway epithelial cells, has been widely described for many years [71,72,217,237–240]. However, these descriptions were based on nonpolarized cells in the majority (S1 Table). Therefore, considering some of the recent findings, this mechanism might be not crucial, and, if observed, only a very small proportion of the bacteria is internalized, depending on bacterial clone and host cell types. The fraction of internalized bacteria in airway epithelium cultured from primary cells cultured at the air–liquid interface was estimated to be very low. From the primary airway cells model, Fleiszig and colleagues found an approximate rate of internalization of approximately 0.0001% within human nasal epithelium or approximately 0.001% in the bovine tracheal epithelium [72]. Conversely, internalization rates in cell lines were higher: approximately 1% to 2% within MDCK or HeLa cells [72,110,138,241] (Fig 5). Furthermore, the host cell membrane composition, the intactness of tight junctions, and the level of cell polarization play a key role in the internalization process. It occurs more frequently on the basolateral cell surface, in cells with disrupted tight junctions or with low polarity levels, the latter being mainly observed on injured or repairing/remodeled epithelium [11,72,73,242–244].

**Internalization process**

As with many other bacteria, *P*. *aeruginosa* internalization involves the formation of membrane lipid rafts [132,245], a signaling platform in the plasma membrane locally enriched in glycosphingolipids and cholesterol, which migrate within the phospholipid bilayer to form lipid aggregates [246]. No lysosomal fusion has been described after vacuole uptake and multiplying bacteria have been observed in the vacuoles [238]. Following bacterial binding, internalization is mediated by the rearrangement of the cellular actin cytoskeleton. The phosphatidylinositol 3-kinase (PI3K) coupled with the Akt pathway is involved in the entry into host cells through both apical and basolateral plasma membranes of epithelial cells [109]. The cytoskeleton microtubules have also been involved in the internalization of the bacteria, via the effectors delivered into cells by the T6SS. This mechanism promotes *P*. *aeruginosa* uptake into epithelial cells by interfering with the PI3K/Akt pathway, through the action of several effectors (H3-T6SS-dependent phospholipase D effector (PldB) and H2-T6SS VgrG2b effector), which interact with the γ-tubulin ring complex [110,216,217,247].

*P*. *aeruginosa* was shown to interfere with the epithelial polarity to enhance binding to the cells: It can induce a subversion of airway epithelial cell polarity to locally transform the apical into a basal plasma membrane and thus allow bacterial attachment and internalization into cells under. After remodeling, the membrane forms protrusions that internalize bacteria without disrupting the tight junctions [248–250].

**Consequences of *P*. *aeruginosa* internalization on the pathogenicity**

The consequences of *P*. *aeruginosa* internalization in airway epithelial cells are still unclear. In an ALI airway epithelium model, which is highly representative of the conditions observed in the in vivo airway epithelium, the extremely low number of internalized bacteria found (around 0.0001%) is likely to indicate a probable accessory role of the internalization in the pathogenesis [72]. Different hypotheses have been proposed.

- *The invasion of the epithelium*. Internalization, as a prerequisite to the invasion of the epithelium, is the most common hypothesis. Some authors argue that internalization, without any killing observed, represents a route for the bacteria to cross the cells, reaches the basal part and the bloodstream system, and allows dissemination to distant organs [109,238]. Thereby, the bacteria could multiply, cross cells to reach the basal part, and invade the surrounding tissues [244,250]. However, all these studies do not define precise mechanisms, and there is no evidence of this transcellular route as the mainstay of its pathogenicity towards the HAE.
- *Persistence and establishment of chronic infections*. As there is no lysosomal killing after internalization, this mechanism has been hypothesized to allow *P*. *aeruginosa* to escape from the different effector cells of the host immune response, such as phagocytes, thereby favoring its persistence and establishment of chronic infections. [217,238,242,250]. Internalization could then induce the transition from an acute to a chronic phenotype in *P*. *aeruginosa* [110].
- *P*. *aeruginosa clearance by airway cell desquamation*. This last and contradictory hypothesis proposes that internalization is more detrimental than beneficial for the bacteria. In healthy individuals, *P*. *aeruginosa* binding to CFTR channels leads to bacterial internalization and triggers host immune system activation, notably inflammation and neutrophil recruitment, which causes the detachment of the infected cells and their removal by mucociliary clearance [251]. Conversely, in CF patients lacking functional CFTR channels, *P*. *aeruginosa* is not internalized, hampering its clearance. The bacteria persist in the dehydrated mucus protected from neutrophils and lead to chronic infections. In this pathology, chronically induced inflammation is ineffective and deleterious [144,237,251].

#### 4) Crossing of the epithelium and dissemination

Recently, a new model has emerged to describe the crossing and the propagation of *P*. *aeruginosa* at the HAE. To gain access to the basement membrane, *P*. *aeruginosa* uses a paracellular route and then radial propagation through the basal compartment (Fig 2C). This new model excludes the suspected, but never demonstrated, theory of a transcellular route used by *P*. *aeruginosa* (by internalization) to cross the epithelium.

Zulianello and colleagues observed the absence of internalization but a paracellular invasion of *P*. *aeruginosa* in differentiated airway epithelia when the integrity of cellular junctions was compromised by rhamnolipids [120]. Subsequently, Heiniger and colleagues demonstrated the exact route used by the bacteria between cells to access the basement membrane during infection [94]. After *P*. *aeruginosa* adhesion to the basolateral surfaces of ciliated cells, the bacteria inject cytotoxic toxins using T3SS (ExoS and ExoT). The toxins lead to cell retraction and detachment, allowing access to other basolateral membranes of adjacent ciliated cells or the resting basal cells, and the progression of *P*. *aeruginosa* through the airway epithelium. Pili-mediated twitching mobility was necessary for this bacterial progression. Radial bacterial dissemination across the entire epithelium was then observed [94]. More recently, Golovkine and colleagues provided a detailed description of this phenomenon. Using a real-time microscopy technique, they described precisely the progression of the paracellular bacterial migration through the epithelium. During the epithelial infection, *P*. *aeruginosa* takes advantage of transient ruptures in the tight junctions to access the basolateral part and once the first bacterium has entered into the breach, a cohort of bacteria rapidly follows at the same entry point. A radial propagation from this entry point through the basal compartment followed, involving the injection of the T3SS-secreted toxins (ExoS and ExoT) into the cells. The pili-mediated twitching motility and the flagella were also necessary for this progression. Interestingly, no internalization of the bacteria was observed in this model, making transcellular migration highly unlikely [93].

## Conclusions

*P*. *aeruginosa* is a major respiratory pathogen, expressing an impressive panel of complexes and complementary virulence factors that, in a well-coordinated manner, promote HAE invasion. However, *P*. *aeruginosa* is an opportunistic pathogen that rarely infects healthy individuals, and the barrier effect of the airway epithelium plays a key role in limiting its pathogenicity. *P*. *aeruginosa* exploits weaknesses in the HAE barrier to gain access to the basolateral part of the epithelium, which is normally inaccessible in healthy epithelium with intact tight junctions. This access is crucial for initiating infection and represents the common point of all the various clinical pathologies due to these bacteria, ranging from lung infections and corneal infections to catheter-related urinary tract infections.

A new pathogenesis model has emerged, replacing a suspected, but incompletely supported, model, where *P*. *aeruginosa* invades through a transcellular route within the epithelium following its internalization. This new paradigm relates to a paracellular crossing of the HAE after access to the basolateral part of the HAE and the radial propagation of *P*. *aeruginosa* through the basal compartment. Therapeutic approaches, including the use of compounds that inhibit access of *P*. *aeruginosa* to the basolateral membrane, could allow alternative treatment strategies, which have the potential to bypass the classic antibiotics, reducing the emergence of antimicrobial resistance.

## Supporting information

S1 Table Models of airway epithelia cells used in the references of the review. ECM, extracellular matrix; ND/NR, not determined/not relevant.
(DOCX)
