# Supplementary material for: Current concepts on Pseudomonas aeruginosa interaction with human airway epithelium
Source: PLoS Pathog. 2023 Mar 30;19(3):e1011221. doi: 10.1371/journal.ppat.1011221 (PMC10062669; doi:10.1371/journal.ppat.1011221)
Supplement: S1 Table — ECM, extracellular matrix; ND/NR, not determined/not relevant. (DOCX) [file ppat.1011221.s001.docx]

Supplemental data

**Table S1: Models of airway epithelia cells used in the references of the review.**

|  | **Species** |  | **Polari-zation** | **Differen-tiation** | **References** |
| --- | --- | --- | --- | --- | --- |
| ***in vivo*** | Human | *tissues* |  |  | 147, 148, 154 |
|  |  | *clinical study* |  |  | 66, 79, 228, 229, 230 |
|  | Mouse |  |  |  | 65, 74, 136, 140, 146, 150, 178, 188, 191, 209, 210, 211, 214, 220, 226, 239, 244 |
|  | Ferret |  |  |  | 65 |
|  | Rat |  |  |  | 182, 197, 248 |
|  | Rabbit |  |  |  | 101, 240, 250 |
|  | Dog |  |  |  | 79 |
|  | Pig |  |  |  | 91, 122 |
|  | C. elegans |  |  |  | 116 |
| ***ex vivo*** | Human | *adenoid tissue* |  |  | 70 |
|  |  | *trachea, bronchi* |  |  | 75, 157 (xenograft) |
|  |  | *nasal mucosa* |  |  | 141 |
|  |  | *nasal brushings* |  |  | 134 |
|  | Rat | *trachea* |  |  | 71 |
|  | Frog | *palate* |  |  | 80 |
|  | Sheep | *nasal brushings* |  |  | 196 |
| ***in vitro*** | Acellular model | *sputum, mucins* |  |  | 85, 88, 89, 90, 91 |
|  |  | *ECM proteins* |  |  | 152, 155, 158, 161, 162 |
|  | Cell lines | *respiratory origin* | - | - | 72, 74, 76, 99, 100, 114, 115, 117, 118, 133, 134, 136, 137, 138, 139, 140, 142, 144, 146, 151, 153, 159, 178, 182, 183, 211, 212, 216, 244, 245, 254 |
|  |  |  | + | - | 73, 74, 76, 115, 116, 120, 123, 136, 144, 149, 151, 160, 199, 212, 213, 237, 246 |
|  |  |  | ND / NR | - | 145, 149, 150, 205, 239, 247, 248 |
|  |  | *other origin* | - | - | 77, 78, 116, 124, 142, 144, 153, 218, 223, 249, 250, 252, 254 |
|  |  |  | + | - | 77, 78, 99, 101, 119, 121, 124, 125, 127, 128, 129, 138, 144, 160, 211, 235, 240, 246, 249, 250, 256, 257 |
|  |  |  | ND / NR | - | 91, 149, 150, 160, 186, 197, 207, 211, 212, 215, 218, 219, 224, 239 |
|  | Primary cells | *respiratory origin* | - | - | 11, 16, 75, 76, 133, 153, 154, 184, 225 |
|  |  |  | + | - | 73, 75, 76, 136 |
|  |  |  | + | + | 11, 16, 75, 100, 121, 126, 184, 225, 238, 248, |
|  |  |  | + | ND / NR | 74, 77, 149 |
|  |  |  | ND | ND / NR | 145, 150 |
|  |  | *other origin* | - | ND/NR | 219 |
|  |  |  | ND/NR | ND/NR | 211 |

ECM: extracellular matrix, ND / NR: not determined / not relevant
